# Conservative management for an esophageal perforation in a patient presented with delayed diagnosis: a case report review of the literature

**DOI:** 10.4076/1757-1626-2-6784

**Published:** 2009-09-15

**Authors:** Konstantinos Tsalis, Konstantinos Blouhos, Dimitrios Kapetanos, Theodore Kontakiotis, Charalampos Lazaridis

**Affiliations:** 1 4^th^ Surgical Department, Aristotle University of ThessalonikiGeorge Papanikolaou Str, Thessaloniki, 57010Greece; 2 Gastroenterological Department, “G. Papanikolaou” General HospitalGeorge Papanikolaou Str, Thessaloniki, 57010Greece; 3 Respiratory Department, Aristotle University of ThessalonikiGeorge Papanikolaou Str, Thessaloniki, 57010Greece

## Abstract

Esophageal perforation is a serious condition with a high mortality rate. Successful therapy depends on the size of the rupture; the time elapsed between rupture and diagnosis, and the underlying health of the patient. Common causes of esophageal perforation include medical instrumentation, foreign-body ingestion, and trauma. A case of esophageal perforation due to fish bone ingestion in a 67-year-old male is described here, with a review of the pertinent literature. The patient presented with chest pain, fever and right-sided pleural effusion. Initial evaluation was nondiagnostic. The water-soluble contrast swallow test showed no evidence of leakage. Computed tomography scan demonstrated a pneumomediastinum, and right-sided hydropneumothorax. The patient was successfully treated using conservative measures.

## Introduction

Esophageal perforation has been regarded as the most serious injury of the digestive tract. Delayed diagnosis and treatment is associated with prolonged morbidity and high mortality [1]. Foreign bodies are common causes of non-iatrogenic esophageal injury [1]. The spectrum of severity can vary from minimal leakage of air in the mediastinum to gross disruption and free drainage into the pleural cavity. Treatment may be conservative or surgical, depending on the cause, site, extent, symptoms, signs, and radiographic findings [1-15]. Today it is accepted that the method chosen for the treatment of esophageal perforation plays an important role in the mortality rate. Therefore, while preserving some well-established principles, therapy must not be confined to narrow boundaries. Each case should be evaluated individually.

## Case presentation

**Figure 1.**
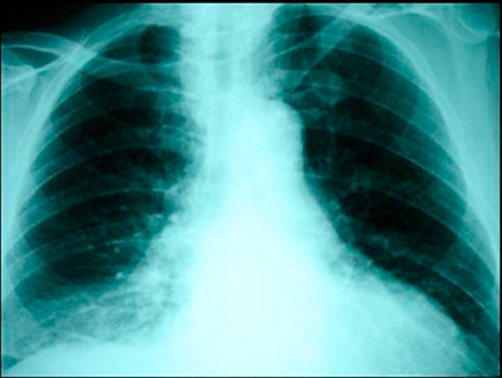
Chest X-ray demonstrated right pleural effusion, but no radio-opacity was detected and there was no evidence of pneumomediastinum or subcutaneous emphysema.

A 67 year old man of Greek origin attended the emergency department with a two hour history of dull central chest pain that radiated into his back. There were no other symptoms and he was normally in good health. Examination and investigations (chest radiography, ECG, full blood count, and biochemistry screen) were thought to be normal. His pain subsided apart from some discomfort on swallowing and he was discharged home. She re-attended the department six days later. He complained that he had been cycling up a hill and had developed severe chest pain radiating into his jaw together with some sweating. Moreover, the discomfort of which he had previously complained had persisted. On examination he had a pulse of 98 per minute, BP 142/72 mm Hg, SaO_2_ 97% on air and temperature 37.5°C. There were no cardiovascular or abdominal signs. There was no surgical emphysema in the supraclavicular fossae. On examination of the chest breath sounds were equal bilaterally for the upper lung fields, but absent for the right lower lung lobe. Chest X-ray confirmed the findings of physical examination and demonstrated right pleural effusion, but no radio-opacity was detected and there was no evidence of pneumomediastinum or subcutaneous emphysema (Figure 1). At this point, a small amount of free air in the right hemithorax was overlooked and the patient admitted to the hospital with the diagnosis questioned for a basal pulmonary pathology.

Because of an erroneous belief that pulmonary complication was the cause of this specific clinical picture, the diagnosis of esophageal perforation was not suspected. The original diagnosis of esophageal perforation was delayed because of misinterpretation of right pleural effusion as a basal pulmonary pathology. Finally, three days after admission clinical deterioration with increased respiratory distress and discomfort, fever and chest pain did arouse suspicion of an esophageal perforation. At this point with a thoroughly history taken, the patient admitted to having had eating fish 12 days ago and the pain begun a few days after (he was attending to Emergency Department three days after), although he had not knowingly swallowed a fish bone.

The investigations were repeated and he now had a raised white cell count (16.3 × 10^3^/ml with a neutrophilia) (reference range, 3.9-10.7 × 10^3^/ml), a somewhat lower haemoglobin concentration (12.8 g/dl previously 14.6 g/dl) and an increased C reactive protein concentration (46 mg/l previously <8 mg/l). The ECG was normal. By this time, the pain was pleuritic and gradually become unbearable. Accordingly, he was given analgesia and high dose intravenous antibiotics. The patient underwent a complementary evaluation, with esophagogram, chest X-ray, and contrast enhanced CT scan tomography revealing a right-sided, distal esophageal rupture, with the coexistence of ipsilateral hydropneumothorax.

**Figure 2.**
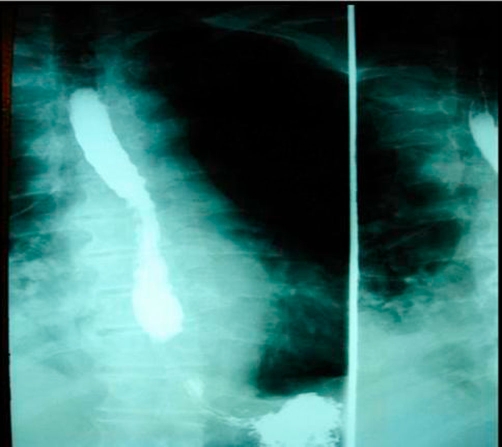
A hypaque swallow study failed to demonstrate extravasation of contrast medium.

**Figure 3.**
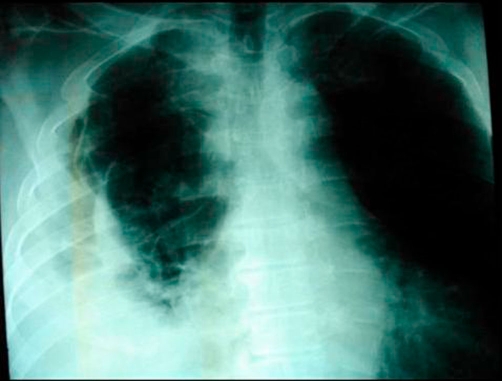
Erect chest X-ray a few hours later demonstrated contrast medium extravasation accompanied with large pleural effusion.

**Figure 4.**
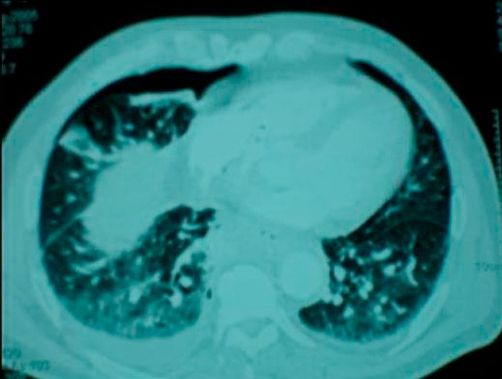
Subsequent CT scan demonstrated right sided pneumothorax, extended right sided pleural effusion and a small amount of air in the mediastinum.

A subsequent hypaque swallow study failed to demonstrate extravasation of contrast medium (Figure 2). Erect chest X-ray a few hours later demonstrated contrast medium extravasation accompanied with large pleural effusion (Figure 3). Subsequent CT scan demonstrated right sided pneumothorax, extended right sided pleural effusion and a small amount of air in the mediastinum (Figure 4).

**Figure 5.**
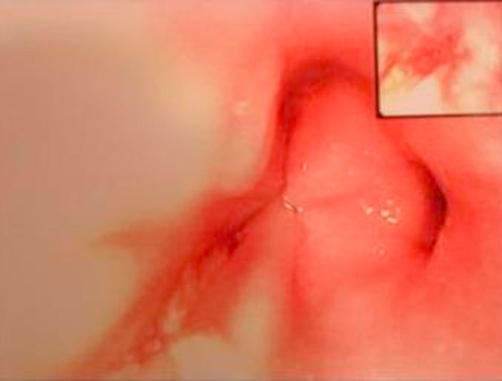
A confirmative esophagogastroduodenoscopy revealed a small distal esophageal perforation.

Furthermore, a confirmative esophagogastroduodenoscopy revealed a small distal esophageal perforation (Figure 5). Fasting was implemented. However, fever subsequently developed (maximum temperature, 38.9^o^C). The white blood cell count was 19.0 × 10^3^/ml. The patient was treated conservatively with intravenous cefuroxime (750 mg every 8 hours), ampicillin (500 mg every 8 hours), and metronidazole (500 mg every 8 hours) to cover the oral bacterial flora.

**Figure 6.**
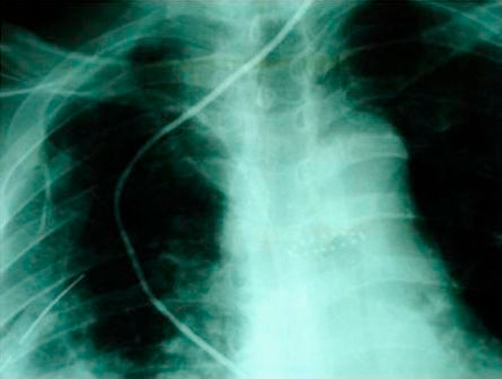
A covered self-expanding metallic stent was inserted endoscopically, across the tear site to prevent ongoing local infection.

A large thoracostomy tube (32 gauge) was immediately placed in close proximity to the rupture site for pleural effusion drainage and the patient was transferred to our surgical unit promptly. A covered self-expanding metallic stent (Ultraflex, Boston Scientific) was inserted endoscopically, across the tear site to prevent ongoing local infection (Figure 6). Oral fluid intake was allowed in increasing amounts and viscosity. Fever decreased rapidly to approximately 38^o^C and subsided after 2 days. The patient’s condition improved and 1 week later there was no leak demonstrated by contrast radiography.

**Figure 7.**
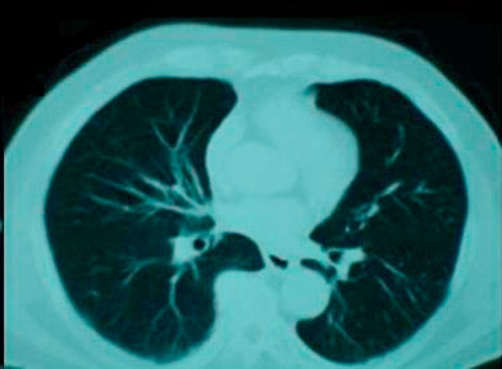
Follow up CT scan at 3 months.

The intravenous antibiotics treatment was discontinued after 5 days, and right-sided chest drain was removed on the 7^th^ day. He recuperated uneventfully and was discharged home 8 days later. The metal stent was removed endoscopically 4 weeks later. Because the stent crossed the lower esophageal sphincter, for the entire treatment time, a high dose of proton pump inhibitors was administered to reduce gastroesophageal reflux. Follow up 3 months after discharge showed the patient to be recovering with no complains (Figure 7).

## Discussion

Foreign bodies can cause esophageal perforation by direct penetration, pressure, chemical necrosis, or during endoscopic removal [1]. They account for 7% to 14% of esophageal perforations [1]. The usual sites affected are the three natural anatomic narrowings: the cricopharyngeus, the crossing of the left main stem bronchus or aortic arch, and the gastroesophageal junction, especially the cricopharyngeus [2].

In a series of 2394 cases of retained esophageal foreign body reported from Hong Kong, perforation occurred in 25 cases (1%) [2]. A wide variety of objects was retained in the esophagus but fish bones were the most common (60%) and chicken bones the second most common (16%). Fish and chicken bones seem to be most commonly associated with major complications, particularly in parts of the world where unfilleted fish is eaten, but other foreign bodies, for example coins, have perforated the oesophagus [3] and fatal esophago-aortic perforation by a coin has been described in a child of three [4]. The diagnosis is frequently missed at initial presentation, as in the case reported here.

There is a tendency for fish bones to migrate and one has been found in the thyroid after perforation of the cervical esophagus, and others in the liver after gastric or gastrointestinal perforation [5]. Foreign bodies most commonly perforate the cervical esophagus [2]. The second most common site for perforation is at the level of the aortic arch [2] where there is scope for fatal or life threatening vascular and respiratory catastrophe, as in the case of a 38 year old man who unknowingly swallowed part of a cocktail stick, which perforated his esophagus and aorta and caused a catastrophic haematemesis 10 days later [6].

Clinical manifestation of foreign-body perforation may be seen immediately or as late as 2 weeks afterwards, as a gradual erosion of the impacted foreign body through the oesophageal wall. The most consistent symptom of an esophageal injury is pain localised along the course of the esophagus [1]. However, up to one third of cases of perforated esophagus are atypical [1]. The most diagnostically useful sign is surgical emphysema. Chest X-rays may show mediastinal and subcutaneous emphysema, pleural fluid, and air. If taken early, the chest X-ray findings can be normal [1].

Mediastinal emphysema can take up to 1 hour to develop, and pleural effusion can take several hours to become evident [1]. Water-soluble contrast esophagography is the diagnostic procedure of choice in patients with clinically suspected perforation of the esophagus, and this test may define the anatomical site and extent of the perforation. False-negative esophagograms occur in 10% to 36% of perforations. Spasm, tissue oedema, and other factors may contribute to false-negative results. Furthermore, leakage may be delayed, so that an immediate esophagogram may fail to demonstrate extravasation [7]. If clinical suspicion of perforation is still high even when the initial esophagogram is negative, another contrast study should be repeated after several hours to demonstrate small tears [7]. Flexible esophagoscopy may miss 20% of injuries. Computed tomography of the chest is more sensitive in detecting mediastinal air and fluid, and may also be useful in cases in which contrast esophagograms cannot be obtained or in cases that are difficult to diagnose or localise. In our case, both first chest X-ray and esophagogram failed to reveal the perforation. The final diagnosis was established after repeated chest X-ray a few hours later and confirmative endoscopy.

Treatment depends on the aetiology, site, and size of perforation; the time elapsed between perforation and diagnosis; underlying esophageal disease; and the overall health status of the patient [8-15]. Small perforations tend to seal without sequelae [1]. Even the injection of methylene blue under pressure can fail to localise the site. Perforation of the cervical esophagus can be managed conservatively in most cases. Perforations of the intrathoracic esophagus that are confined to the mediastinum can be adequately treated using conservative measures in most patients [1].

Criteria for non-surgical treatment include perforation that is confined to the mediastinum, drainage of the cavity back into the esophagus, clinical stability, and minimal clinical signs of sepsis [14-15]. Perforations of the lower two thirds of the esophagus that affect the pleura, pericardium, or peritoneum require rapid surgical intervention [15].

In contrast to the surgical approach, a nonoperative treatment regime was mainly used for patients unsuitable for surgery. In the past, conservative treatment was limited to antibiotics, insertion of a nasogastric tube, acid suppression, and nothing by mouth. Recently, encouraging results were reported about the sealing of esophageal perforations by insertion of endoluminal prosthesis. The majority of reported cases demonstrate that the main principles of the surgical treatment, namely, the rapid closure of the esophageal leak and drainage, can also be achieved by minimal invasive endoscopic approach by inserting a covered metal stent, followed by interventional drainage.

As reported by others [7], there is a strong correlation between the elapsed time between onset of esophageal perforation and treatment. With an increased delay between perforation and treatment, the prognosis worsens owing to the establishment of sepsis and progressive organ failure. With regard to time of endoscopic management, in our case, it took much longer than 24 hours to be offered. To better assess the inflammatory status, we suggest not only to pay attention to the “classical” time gap between perforation and diagnosis but also to the aetiology and status of the inflammatory response, according to clinical and laboratory examinations. In addition to these clinical findings, a CT scan of the chest is recommended whenever Esophageal Perforation is suspected. In our case, nonoperative management was chosen, based on the fact that patient’s general condition was not impaired and progressive sepsis was not apparent.

Based on this obvious clinical correlation, we note that the primary goal of any treatment of an esophageal perforation should be that the wall defect be sealed as soon as possible. In the case of an instrumental perforation, the stent should be inserted during the same procedure [8,9]. It is recommended the Ultraflex stent in the case of an acute esophageal perforation because of its very fast and complete expansion [10]. With this approach, the perforation can be sealed immediately, which consequently prevents sepsis and organ failure because of minimal contamination of the mediastinum and pleural cavity. In case of an old esophageal perforation, a fast stent expansion is less vital because contamination has already taken place. Therefore, it is recommended a totally covered Niti-S-Stent, which expands more slowly but could be easily extracted after weeks or even months. In old perforations with an extended wall defect and a contaminated pleural cavity, additional thoracoscopic irrigation and wide drainage might be advisable.

Stent extraction after healing should always be performed because severe stent complications after long-stay treatment are well documented [12]. The exact period during which the stent should be in place for complete healing is still unknown. Segalin and coworkers [11] removed the tube after 2 to 3 weeks, whereas Dorman and associates [9] reported a period of 4 months for a self-expanding stent. Siersema and coworkers [13] retrieved stents after a median of 7 weeks after application. In general, it is recommended a period of 10 days for small esophageal perforations and as long as 8 weeks for extended oesophageal wall defects. If the stent crosses the lower oesophageal sphincter, early extraction is vital because there is a high risk of gastric acid reflux, which in the worst case may provoke aspiration pneumonia. In those cases, a high dose of proton pump inhibitors is necessary to reduce the amount of gastric success. Completely covered stints are easy to extract even after months. Partially covered Ultra flex stints preferably should be removed within 4 weeks because the mucosa grows through the no covered part, and extraction might cause a partial mucosectomy with bleeding and consecutive stenosis of the esophageal lumen. On the other hand, an advantage of partially covered stents is that the stent is less likely to migrate.

Stent removal after healing should always be performed and is not associated with increased morbidity or mortality. Primary repair of esophageal perforations is still considered the “gold standard” [14], but the encouraging results among early treated patients may be a fertile foundation for changing this paradigm, at least for patients treated early.

The general consensus is to identify the clinical problem quickly, for timely clearance of the inflamed esophageal focus. The optimal approach to esophageal perforation remains problematical and controversial [15]. Each case should be evaluated individually. Nonoperative management can be easily applied in carefully selected cases. Early recognition and commencement of treatment is of paramount importance and this is possible only if a high index of suspicion is maintained in these patients.

## Conclusion

From this case of esophageal perforation, it can be concluded that plain X-ray cannot rule out the presence of a foreign body in the oesophagus. Early endoscopy is needed if clinical suspicion of an impacted foreign body is high. Small pneumomediastinum may not be detectable on the chest X-ray, and small oesophageal perforations may not be detectable by performing a water-soluble contrast study. The present report demonstrates that a minimal invasive treatment approach of a self-expandable metal stent insertion is a justified and safe method for sealing esophageal perforations. Even in cases of old esophageal perforations as in our case, sealing with self-expandable metal stents achieves an excellent outcome. Additional thoracoscopic irrigation and drainage might be advisable in case of extensive thoracic cavity contamination.
